# Transumbilical single-site two incision laparoscopic pyloromyotomy for pediatric hypertrophic pyloric stenosis

**DOI:** 10.1186/s12893-022-01672-2

**Published:** 2022-06-07

**Authors:** Yi Ji, Xiaoqin Lai, Zhicheng Xu

**Affiliations:** 1 Department of Pediatric Surgery, West China Hospital of Sichuan University. #37 Guo-Xue-Xiang, Chengdu, 610041 Sichuan China; 2 Day Surgery Center, West China Hospital, Sichuan University, Chengdu, 610041 Sichuan China; 3 West China School of Nursing, Sichuan University, Chengdu, 610041 Sichuan China; 4 China International Emergency Medical Team (Sichuan), Sichuan University, Chengdu, 610041 Sichuan China

**Keywords:** Hypertrophic pyloric stenosis, Transumbilical laparoscopic pyloromyotomy, Minimally invasive method, Children, Follow-up

## Abstract

**Purpose:**

A new novel technique for the treatment of pediatric hypertrophic pyloric stenosis (HPS), transumbilical single-site laparoscopic pyloromyotomy with a single instrument (TUSSLP), was introduced. TUSSLP was compared with the transabdominal three-site laparoscopic pyloromyotomy (TATSLP) procedure.

**Methods:**

Patients with HPS who underwent TUSSLP and TATSLP between January 2016 and September 2020 were assigned to group A and group B, respectively. The descriptive variables, perioperative clinical characteristics and postoperative follow-up results were retrospectively analyzed and compared between the 2 groups. The primary outcome of this study was the rate of switching to conventional pyloromyotomy.

**Results:**

Sixty-four patients were enrolled in this study. Of these patients, 29 (22 males, 7 females, 54.4 ± 22.6 days) who received TUSSLP were assigned to group A. The remaining 35 (28 males, 7 females, 54.5 ± 27.6 days) who received TATSLP were assigned to group B. The data of preoperative patient variables were comparable between the 2 groups (*P* > 0.05). The mean operative time (ORT) was 28.1 ± 5.6 min in group A, which was not significantly different from 25.8 ± 3.1 min in group B (*P* = 0.25). The other perioperative features were not significantly different between the 2 groups (*P* > 0.05). During follow-up (39.1 ± 14.7 m in group A and 35.4 ± 16.1 m in group B, *P* = 0.51), no significant difference was observed in the overall incidence of vomiting between the 2 groups (*P* = 0.26).

**Conclusions:**

TUSSLP is a feasible and reliable minimally invasive method for HPS. It has the advantages of an improved cosmetic appearance. The postoperative follow-up results of TUSSLP are comparable with those of TATSLP.

## Introduction

Infantile hypertrophic pyloric stenosis (HPS) is a common cause of vomiting during the neonatal period, with an incidence of approximately 2 per 1000 live births[1]. In patients with HPS, symptoms usually occur between 3 and 8 weeks of life, with forceful or projectile nonbilious vomiting after feeding. Patients may suffer from weight loss, dehydration and alkalosis [2]. Demian et al. showed that only 5.8% of infants diagnosed with HPS were less than 14 days old. Patients presenting in the first 2 weeks of life had a significantly higher positive family history for HPS than infants who presented with HPS after day 14 of life [3]. In addition to the early presentation of HPS, late presentation of HPS at the 14th week of life has been reported in the literature as a rare event [4].

The first laparoscopic pyloromyotomy (LP) procedure was reported in 1990 by Alain et al. [5]. With the advancement of laparoscopic instrumentation for infants, this technique has gained popularity [6–10]. Compared with laparotomy, LP has a shorter hospital stay, lower morbidity and lower risk of major wound-related complications. However, placing three trocars through a small umbilicus can have the disadvantage of conflicting instruments, so only a few pediatric centers can handle this technique [11, 12]. In addition, incomplete pyloromyotomy and mucosal perforation have been reported in patients receiving LP. In the majority of the centers, traditional LP is performed using a retractable pyloromyotomy knife for incision of the hypertrophied pylorus. Most recently, the use of a hook with electrocautery was reported with excellent outcomes [13]. Since January 2017, we have attempted a novel procedure of transumbilical single-site laparoscopic pyloromyotomy with a single instrument (TUSSLP) through only two incisions around the umbilical ring. In this report, we described the distinct features of TUSSLP.

## Materials and method

### Design and study population

This was a retrospective study of patients with HPS who underwent TUSSLP and transabdominal three-site laparoscopic pyloromyotomy (TATSLP) between January 2016 and September 2020. Approval was obtained from the West China Hospital of Sichuan University Institutional Review Board (NO. 2016-118). All procedures followed the research protocols approved by Sichuan University and West China Hospital of Sichuan University and were conducted according to the Declaration of Helsinki. Written informed consent was provided by the patients’ parents for their clinical records to be used in this study.

The diagnosis of HPS was based on the history, palpation of a hypertrophied pyloric muscle and ultrasonography. Patients’ parents were given the option to choose the treatment (either TUSSLP or TATSLP). The inclusion criteria were as follows: patients with clinically confirmed HPS and patients who received either TUSSLP or TATSLP. The exclusion criteria were as follows: patients with comorbidities and patients who failed to attend the follow-up. The patients receiving TUSSLP and TATSLP were assigned to group A and group B, respectively.

### Study outcomes

The data of perioperative patient characteristics and postoperative follow-up results were retrospectively analyzed and compared between the 2 groups. The primary outcome of this study was the rate of switching to conventional pyloromyotomy. Secondary outcomes included operative time (ORT), duration of anesthesia, and intraoperative and postoperative complications. We hypothesized that patients receiving TUSSLP might have a comparable rate of switching to conventional pyloromyotomy and postoperative complications with those receiving TATSLP.

## Surgical technique

### The TUSSLP procedure

**Fig. 1:**
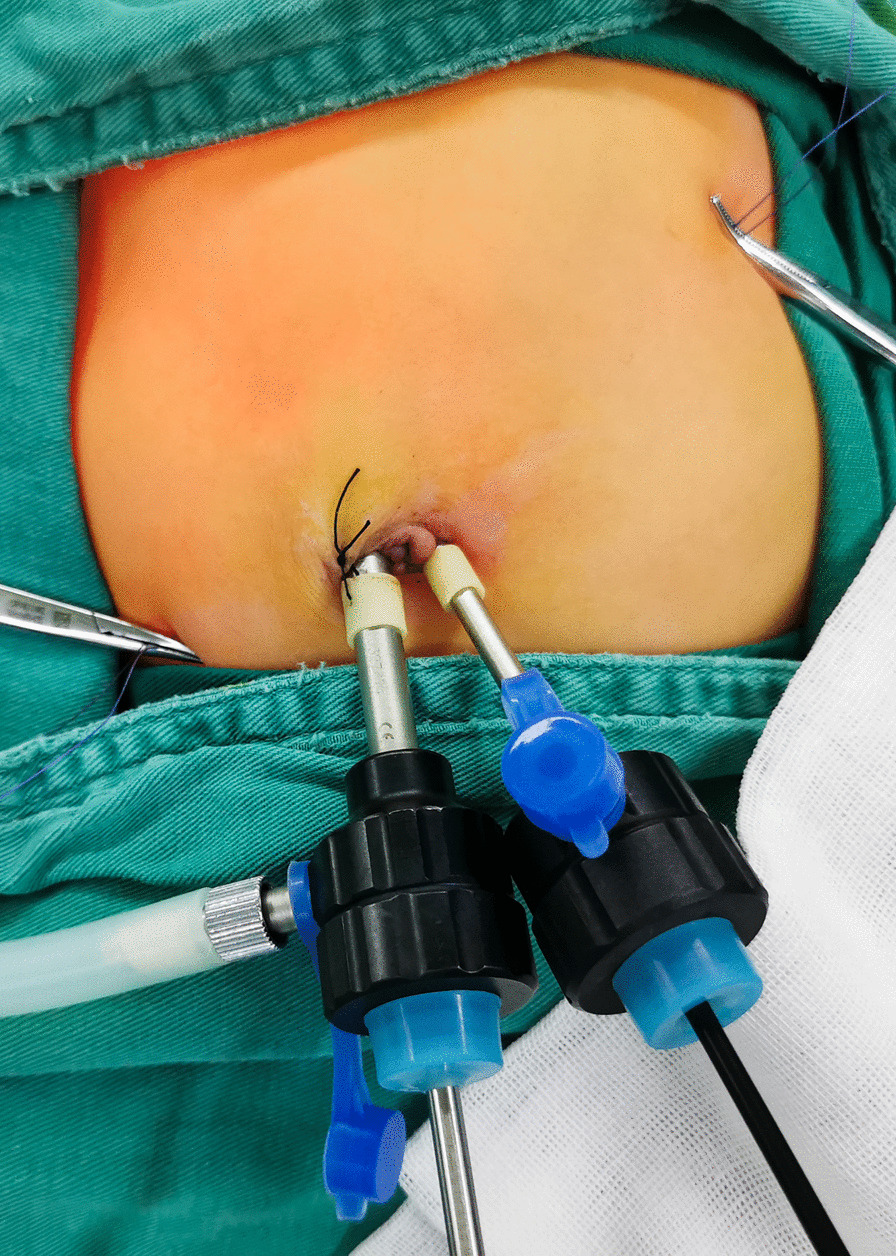
A 5 mm incision was made at the right rim of the umbilical ring for a 5 mm trocar and a 30° laparoscope. The second 3 mm incision was made at the left rim of the umbilical ring for a 3 mm trocar and the related 3 mm instruments

**Fig. 2:**
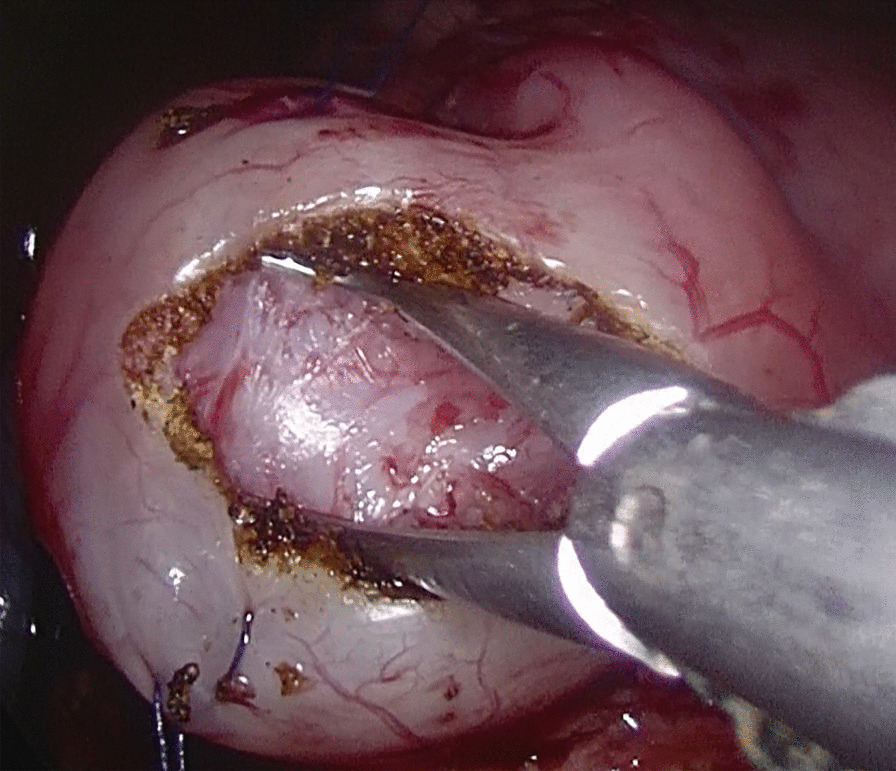
The Maryland forceps were used to divide the wound of pyloric muscle fully to make the mucous membrane completely bulge

**Fig. 3:**
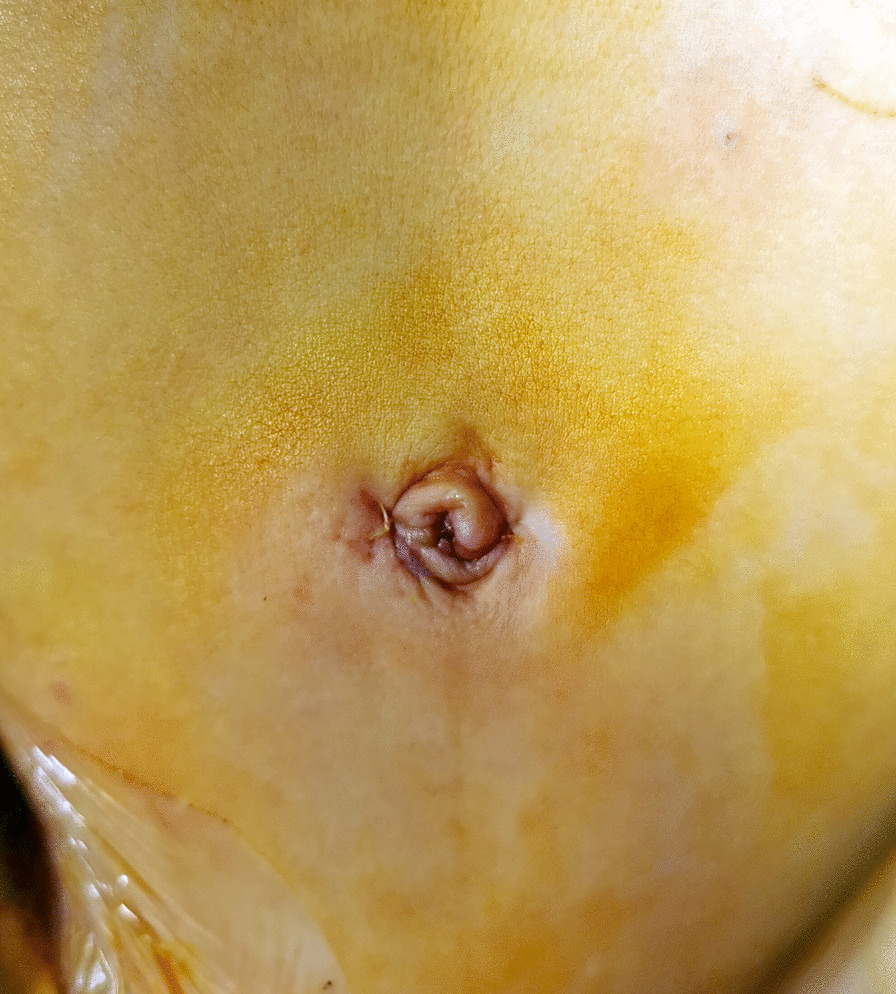
The two incisions (right 5 mm, left 3 mm) on the bilateral rim of the umbilical ring were closed with 5-0 Vicryl thread

In the TUSSLP procedure, the patient was placed in a supine position with a monitor at the patient’s head. The operator stood on the left side of the patient’s feet, and the camera assistant stood on the other side. A 5 mm incision was made through the right rim of the umbilical ring with the open Hasson technique to establish the pneumoperitoneum at a pressure of 6–8 mmHg with a flow rate of 3–6 L/min. A 5 mm incision was made at the right rim of the umbilical ring for a 5 mm trocar and a 30° laparoscope. The second 3 mm incision was made at the left rim of the umbilical ring for a 3 mm trocar and the related 3 mm instruments. (Fig. 1). Laparoscopy was started by inspection of the pyloric olivary mass and further confirmed the diagnosis. The needle swaged with 4–0 Vicryl thread punctured into the peritoneal cavity at the left upper quadrant and right lower quadrant of the abdominal wall leaving the thread end outside. Under control with the laparoscopic needle holder only, the needle snaped the superior and inferior margins of the pyloric tube and pierced back to the outside abdominal wall from the position of entering the peritoneal cavity individually. Two percutaneous sutures were introduced separately to hold the pylorus in place. The pyloric canal could be secured firmly by simultaneously tensioning both external thread ends at the left upper quadrant and right lower quadrant of the abdominal wall. The nonvascular area on the anterior wall of the pyloric tube was cut longitudinally with a monopolar electric hook through the 3 mm trocar at the left rim of the umbilical ring. Then, 3 mm Maryland forceps were used to fully divide the wound of pyloric muscle to make the mucous membrane completely bulge (Fig. 2). Fifty milliliters of air was slowly injected into the gastric tube, and a few drops of saline were poured through the port to check for any inadvertent mucosal injury. If not, the air was evacuated. After confirming that there was no active bleeding from the edge of the pylorus muscle, the instruments were removed and pneumoperitoneum was exsufflated. The incisions on the bilateral umbilical rim were closed with 5–0 absorbable thread (Fig. 3).

### The procedure of TATSLP

In the TATSLP procedure, the patient position was the same as that in the TUSSLP procedure. A 3 mm incision was made through the center of the umbilicus with the open Hasson technique to establish the pneumoperitoneum at a pressure of 6–8 mmHg with a flow rate of 3–6 L/min. A 5 mm trocar and a 30° laparoscope were introduced into the peritoneal cavity. Under laparoscopy, the two 3 mm trocars were placed in the bilateral upper quadrant of the abdominal wall. Laparoscopy was started by inspection of the pyloric olivary mass and confirmed the diagnosis. Through the trocar on the right upper quadrant of the abdominal wall, the undamaged forceps were placed at the ear of the pyloric tube to catch the duodenum. Through the trocar on the left upper quadrant of the abdominal wall, the pyloric knife was inserted. From the end of the duodenum to the stomach, the nonvascular area on the anterior wall of the pyloric tube was cut longitudinally. Then, the Maryland forceps were used to fully separate the wound of pyloric muscle to make the mucous membrane completely bulge. The remaining surgical steps were the same as those in the TUSSLP procedure.

### The postoperative feeding regimen

The protocol of the postoperative feeding regimen was the same for each group. Feeds were started at 8 h postoperatively. The initial feeding was 20 mL of water; if this was well tolerated, the infant could have 50 mL 2 h later and formula feeding 2 h later. When vomiting occurred, the next feeding was omitted and was resumed 6 h later at the same dose. The baby was adequately discharged after 24 h of ad libitum feeding. All episodes of vomiting were recorded.

### Data collection and statistical analysis

First, all patients were analyzed for their perioperative clinical features by reviewing the medical charts. Second, the follow-up data were collected using a telephone questionnaire or the last visit to our outpatient clinic according to the medical files.

We used descriptive statistics to compare characteristics between the two groups: frequencies with percentages for qualitative variables and means with SD for continuous variables. Comparisons of characteristics were constructed with Student’s *t* test for continuous variables where appropriate and Fisher’s exact test or a chi-square test for categorical variables. The software applied for statistical calculation was IBM SPSS 22.0 for Windows 10.0 (IBM Corp.) A P value < 0.05 was considered statistically significant.

## Results

### Data on perioperative patient characteristics

There were 74 patients with HPS who received TUSSLP or TATSLP between January 2016 and September 2020. Ten patients were excluded from the study. Of them, 7 patients had comorbidities (in the TUSSLP group, 3 patients had inguinal hernia and 1 patient had malrotation; in the TATSLP group, 2 patients had inguinal hernia and 1 patient had cryptorchidism), and 3 patients were lost to follow-up (1 patient in the TUSSLP group and 2 patients in the TATSLP group). The remaining 64 cases were enrolled in this study. Of them, 29 patients receiving TUSSLP were assigned to group A (22 males, 7 females, 54.4 ± 22.6 days), and 35 patients receiving TATSLP were assigned to group B (28 males, 7 females, 52.5 ± 27.6 days). The preoperative patient variables, including age, sex, weight, pyloric muscle thickness and length measured by ultrasonography, and the duration of symptoms were not significantly different between the 2 groups (*P* > 0.05) (Table 1).

**Table 1:** Preoperative patient characteristics

Characteristics	Group A (n = 29)	Group B (n = 35)	*P*
Age (days)	54.4 ± 22.6	52.5 ± 27.6	.35
Sex (males)	22	28	.69
Weight (kg)	3.9 ± 0.6	4.1 ± 0.5	.84
Pyloric muscle thickness (mm)^a^	4.6 ± 1.0	4.2 ± 0.8	.25
Pylorus length (mm)^a^	20.8 ± 2.1	21.3 ± 2.3	.72
Chloride	103.1 ± 5.4	102.8 ± 7.1	.86
Bicarbonate	27.1 ± 4.3	26.7 ± 5.4	.73
Sodium	137.6 ± 3.2	139.1 ± 4.3	.56
Duration of symptoms (days)	10 (4–40)	9 (7–35)	.23

Quantitative data are expressed as the mean ± SD or median (25th-75th percentile), and categorical data are expressed as a number (percentage)

^a^Measured by ultrasonography

During the operation, most patients did well in both groups. There was 1 case in group A and 2 cases in group B who switched to conventional pyloromyotomy due to mucosal perforation (2 patients) and duodenal injury (1 patient). The mucosal perforations and duodenal injury were emphasized intraoperatively and were managed by an umbilical open procedure. The ORT was 28.1 ± 5.6 min in group A, which was not significantly different from 25.8 ± 3.1 min in group B (*P* = 0.25). The other perioperative patient characteristics, including the duration of anesthesia and intraoperative complications (mucosal perforation, duodenal injury), were not significantly different between the 2 groups (*P* > 0.05) (Table 2).

**Table 2:** Intra- and postoperative patient characteristics

	Group A (n = 29)	Group B (n = 35)	*P*
Operative time (min)	28.1 ± 5.6	25.8 ± 3.1	.25
Duration of anesthesia (min)	95.3 ± 10.1	84.5 ± 5.9	.34
Intraoperative complications	1 (3.4%)	3 (8.6%)	.40
Mucosal perforation	1 (3.4%)	1 (2.9%)	.89
Duodenal injury	0 (0.0%)	2 (5.8%)	.19
Conversion	1 (3.4%)	2 (5.8)	.67
Postoperative complications	2 (6.8)	3 (8.6%)	.80
Wound infection	1 (3.4%)	2 (5.8%)	.67
Wound dehiscence	0 (0.0%)	1 (2.9%)	.36
Revision of pyloromyotomy	1 (3.4%)	2 (5.8%)	.67
Time to full feeding (h)	38.3 ± 5.6	34.5 ± 8.6	.59
Postoperative length of stay (days)	3.8 ± 1.2	3.6 ± 2.7	.65

Quantitative data are expressed as the mean ± SD, and categorical data are expressed as a number (percentage)

### Postoperative results

The response rate for the telephone questionnaire or clinic interview for the patients enrolled in this study was 95.5%, including 29/30 patients in group A (96.7%) and 35/37 patients in group B (94.6%). The data of 3 patients (1 after TUSSLP, 2 after TATSLP) who had incorrect phone numbers or no family member was contactable for the telephone or clinical interview were not collected.

The follow-up time was 39.1 ± 14.7 months in group A and 35.4 ± 16.1 months in group B (P = 0.51). The postoperative complications, including wound infection, wound dehiscence, revision of pyloromyotomy, time to full feeding, and postoperative length of stay, were not significantly different between the 2 groups (*P* > 0.05) (Table 2). The overall incidence of vomiting was also not significantly different between the 2 groups (15 versus 23, *P* = 0.26). All the vomiting symptoms of patients among the 2 groups disappeared after conservative treatment.

## Discussion

With the emergence of scarless operations involving the abdominal wall, single-site umbilical LP has also been reported [12, 14–16]. However, single-site umbilical LP is difficult to perform in newborns and has a high probability of severe complications, such as mucosal perforation and recurrent obstruction [17]. Therefore, single site umbilical laparoscopy has not been greatly reported in recent years. Based on many years of experience with TATSLP, the TUSSLP procedure was developed to overcome many difficulties with transumbilical single-site LP. Here, we present our first 29 cases of HPS who underwent TUSSLP. The majority of patients in our study had the expected excellent outcomes associated with this disease. We found that the TUSSLP procedure is a feasible and reliable minimally invasive method for patients with HPS.

HPS usually presents at 2 to 6 weeks of age in previous healthy infants. Increased awareness of HPS is required for early diagnosis and prompt treatment to prevent potential complications, although there is evidence suggesting that duration of the symptoms (e.g., vomiting duration) at presentation did not have a significant impact on postoperative outcomes. Our patients (mean ages, 50–52 days) were older than those patients reported in a previous study (mean ages, 30–40 days) [2, 18]. The delayed diagnosis of our patients may be due to the unawareness of HPS before referral. We were not surprised to find that our patients had a long duration of preoperative vomiting. Preoperative weight may have a significant impact on mucosal perforation and weight loss. Aurélien et al. proposed that low weight might be correlated with a lower amount of workspace for laparoscopy [18].

Kozlov et al. [19] suggested that an endoscope longer than the other instruments could help the assistant’s hands out of the operator working space, and the angulation of the optical axis of at least 30° provides an offset, rather than inline, view of the pylorus. To resolve the problems above, we developed the TUSSLP procedure, in which the LP was performed through two incisions around the umbilical ring. The pyloric canal can be firmly secured and fully exposed by simultaneously tensioning both external thread ends at the left upper quadrant and right lower quadrant of the abdominal wall [13, 20].

Common perioperative complications after pyloromyotomy include incomplete pyloromyotomy and mucosal perforation [8, 21–23]. In the present study, satisfactory TUSSLP results were achieved in our patients. Our findings were consistent with other published series that reported an incidence of incomplete myotomy of 2% to 8% [8, 9, 13] and mucosal perforation of 1.3 to 5.0% [8, 24]. Our overall conversion rate of 4.7% was comparable to those reported by previous studies [2]. The conversions in our study series were due to mucosal perforations and duodenal injury. This relatively higher conversion rate might be attributed to the learning time and complete technique acquisition of LP. Oomen et al. demonstrated that the procedure acquirement for LP reveals a trend toward the reduction of postoperative complications after 35 procedures, suggesting that the first 35 patients might reflect the learning curve [23]. There is also evidence suggesting that the LP procedure has a steep learning curve for novices [25–27], especially in the management of TUSSLP for the first 10–20 cases [26]. Thus, surgical teaching using simulators for residents or younger consultants is highly advisable, and it could in fact be deemed to be crucial for the safe performance of LP [28].

In the literature, wound infection, fascial dehiscence and omental herniation are frequently reported postoperative complications. Previous studies found that there were no significant differences in the postoperative complication rate between open and laparoscopic pyloromyotomy [18, 21]. In the present study, no severe postoperative complications were documented in either of the approaches. The overall postoperative complication rate of LP in our cohort was 7.8%, which was comparable to that in previous studies [8, 21, 23]. When comparing the rate of the most common postoperative complication, our results are equivalent to others, with a rate of 4.7% for wound infection in our cases. None of our patients had incisional hernias or inflammatory scars, both of which can be seen in open pyloromyotomy. During the follow-up, the overall incidence of vomiting after TUSSLP was not significantly different from that after TATSLP. The vomiting subsided without requiring any further medication. Our total vomiting rate is relatively high compared to others. However, many authors did not report postoperative vomiting in their studies. A possible cause of the relatively high vomiting rate is that the age at the time of operation in our cohort was higher than that in others. We cannot exclude referral or selection bias as the reason for this phenomenon.

Limitations of the current study should be admitted. First, this was a retrospective study with a single-center design. The study site is a tertiary referral center, adding potential selection bias toward more severe patients. Second, the 10 cases of HPS excluded from the study might reverse the results of the statistical comparison between the 2 groups. Although this study provided standard items for comparison between two procedures, it mostly affected comparison of the cosmetic, which resulted in favor of TUSSLP. Third, due to the small number of patients included in the study, we could not perform a risk analysis for complications. Further studies on a larger number of cases may be required for more accurate conclusions.

## Conclusion

We conclude that there are no differences between the TUSSLP and TATSLP techniques in terms of the ORT, perioperative complications, conversion to open pyloromyotomy, time to resumption of feeding, duration of hospitalization and postoperative follow-up results. Although TUSSLP surgery is complex and demanding, it is still regarded as a valid alternative to the classic TATSLP procedure with identical clinical results and better esthetic appearances for an experienced surgeon. The TUSSLP procedure is a feasible and reliable minimally invasive method for HPS in well-trained hands.

## Data Availability

The datasets used and/or analyzed during the current study are available from the corresponding author on reasonable request.
